# Promoter Cis-Element Analyses Reveal the Function of αVPE in Drought Stress Response of *Arabidopsis*

**DOI:** 10.3390/biology12030430

**Published:** 2023-03-10

**Authors:** Chu-Nie Tang, Wan Muhamad Asrul Nizam Wan Abdullah, Chien-Yeong Wee, Zetty Norhana Balia Yusof, Wai-Sum Yap, Wan-Hee Cheng, Nadiya Akmal Baharum, Janna Ong-Abdullah, Jiun-Yan Loh, Kok-Song Lai

**Affiliations:** 1 Department of Cell and Molecular Biology, Faculty of Biotechnology and Biomolecular Sciences, Universiti Putra Malaysia, Serdang 43400, Selangor, Malaysia; 2 Biotechnology and Nanotechnology Research Centre, Malaysian Agricultural Research and Development Institute, Serdang 43400, Selangor, Malaysia; 3 Department of Biochemistry, Faculty of Biotechnology and Biomolecular Sciences, Universiti Putra Malaysia, Serdang 43400, Selangor, Malaysia; 4 He & Ni Academy, 1-3, The Boulevard, Mid Valley City, Lingkaran Syed Putra, Kuala Lumpur 59200, Selangor, Malaysia; 5 Faculty of Health and Life Sciences, INTI International University, Persiaran Perdana BBN, Putra Nilai, Nilai 71800, Negeri Sembilan, Malaysia; 6 Centre of Research for Advanced Aquaculture (CORAA), UCSI University, Cheras, Kuala Lumpur 56000, Selangor, Malaysia; 7 Health Sciences Division, Abu Dhabi Women’s College, Higher Colleges of Technology, Abu Dhabi 41012, United Arab Emirates

**Keywords:** drought, motifs, stress, vacuolar processing enzyme

## Abstract

**Simple Summary:**

Vacuolar processing enzyme (VPE) is a cysteine protease responsible for vacuolar proteins’ maturation and regulation of programmed cell death in plants. Among four isoforms of *Arabidopsis thaliana* VPEs, only the functions of βVPE, γVPE, and δVPE were determined. Hence, to investigate the possible function of αVPE, promoter analysis, co-expression network, gene expression profiling, and loss of function studies were performed. Repetitive drought-related cis-elements such as ABRE, MBS, MYC, and MYB were successfully identified with the aid of PlantCARE and PLACE databases. Similarly, the co-expression network also revealed that genes interacting with *αVPE* were involved in drought-regulation-related function. In addition, *A. thaliana* under drought treatment recorded an upregulation of *αVPE* expression (2.7-fold). Loss of function study through *αvpe* knockout mutants showed that *αvpe* mutants remained viable with 22% higher water retention as compared with wild-type after drought treatment. Biochemical analyses recorded a 47% reduction in proline activity, 70% decrease in sucrose content, and 39% lower MDA content, but 50% increased photosynthetic pigments in *αvpe* mutants. Altogether, our study provided important proof and a foundation for understanding the involvement of αVPE in modulating drought tolerance in *A. thaliana*.

**Abstract:**

Vacuolar processing enzyme (VPE) is a cysteine protease responsible for vacuolar proteins’ maturation and regulation of programmed cell death (PCD). Four isoforms of *Arabidopsis thaliana* VPEs were identified previously, but only the functions of βVPE, γVPE, and δVPE were determined. The specific function of a gene is linked to the cis-acting elements in the promoter region. A promoter analysis found repetitive drought-related cis-elements in αVPE, which highlight its potential involvement in drought regulation in *A. thaliana*. The further co-expression network portraying genes interacting with *αVPE* substantiated its drought-regulation-related function. Expression of *αVPE* was upregulated after drought treatment in *A. thaliana*. To confirm the role of αVPE, a loss of function study revealed that αVPE knockout mutants remained green compared with WT after drought treatment. The mutants had reduced proline activity, decreased sucrose content, and lower MDA content, but increased photosynthetic pigments, indicating that αVPE negatively regulates drought tolerance in *A. thaliana.* Taken together, our findings serve as important evidence of the involvement of αVPE in modulating drought tolerance in *A. thaliana*.

## 1. Introduction

A gene promoter region is normally about 1000 bp upstream of the transcription start site of a gene coding sequence [1]. The cis-elements in the promoter region help plants to react to environmental changes by navigating the regulation of corresponding downstream genes at the transcriptional level [2,3]. Generally, cis-elements are specific short DNA motifs range from 5 to 25 bp in length [3,4]. Identification of these cis-elements helps to predict its regulatory role at the transcriptional level. MYB elements in *Arabisopsis thaliana* were reported to be involved in the response to dehydration [1]. Light-response elements such as I-Box and AE-Box exist in the promoter of *ZmRXO1* and might be involved in the light induction mechanism in maize plants [5]. Many stress-resistant genes that regulate downstream genes’ expression under abiotic stresses in grapevine plants were found to have ABA-responsive elements (ABREs) [2]. Therefore, a better understanding of promoter sequence together with the number, type, and distribution of cis-elements will aid in revealing the specific functions of a gene in plants [2].

Vacuolar processing enzyme (VPE) is a cysteine proteinase [6] first discovered in *A. thaliana* to be responsible for the maturation of seed storage proteins and programmed cell death (PCD) [7,8]. To date, four isoforms of *A. thaliana* VPEs (*At*VPEs), which are αVPE, βVPE, γVPE, and δVPE, have been identified [9]. Studies revealed that βVPE and δVPE were upregulated during basal defenses of *A. thaliana* against the phloem-feeding insect [10]. Besides, γVPE was found to be upregulated during phytohormone treatment, oxidative stress, heavy metal, and abiotic stresses via the promoter–reporter fusion approach [7]. However, to date, the function of αVPE remains unknown. In this study, promoter analysis was performed to predict the possible function of αVPE with the aid of PlantCARE and PLACE databases. The cis-elements in the promoter of *αVPE* were identified and analyzed, and a co-expression network on *αVPE* was constructed to further understand the function of the co-expressed genes. The expression profile of the *AtVPE*s family obtained following drought treatment showed that *αVPE* and *γVPE* were upregulated. Further loss of function was performed using αVPE knockout mutants subjected to drought treatment and biochemical assay validation. Our results provide significant proof of the involvement of αVPE in drought regulation.

## 2. Materials and Methods

### 2.1. Plant Materials and Growth Conditions

Seeds of wild-type (WT) *A. thaliana* ecotype Columbia (Col-0) and αVPE-3 knockout mutant (alpha mutant) accessions with T-DNA insertion (CS67914), in the Col-0 background, were obtained from ABRC (*Arabidopsis* Biological Resource Center) and sown on soil. The seedlings were grown in a growth chamber under a constant light of approximately 100 µmol·mol^−2^·s^−1^ with a 16 h light/8 h dark photoperiod and 50–70% humidity. Mutant confirmation was performed by PCR with LB (5′-TAGCATCTGAATTTCATAACCAATCTCG-3′), BP (5′-CGAAGCTTATGCCAGAA-ATGGACAA-3′), and RB (5′-CAAACTAGGATAAATTATCGCGCGCGGTGTCA-3′) primers (see Figure S2).

### 2.2. Drought Treatment

Thirty-day-old seedlings for both WT and alpha mutants were subjected to drought treatment by withholding water for 7 days in a greenhouse at 22 °C, 70% relative humidity, and a 16 h light/8 h dark photoperiod. The leaves were then sampled, frozen in liquid nitrogen, and stored at −80 °C until further use.

### 2.3. Promoter Sequence Analysis

The 2500 bp upstream sequence of *αVPE* gene (NCBI Reference Sequence: NC_003071.7) was selected as the promoter sequence and submitted to PlantCARE [11] and PLACE Web Signal Scan [12] for cis-element prediction analysis.

### 2.4. Co-Expression Network Modeling

Co-expression network models of *αVPE* gene were generated using ATTED-II version 6.1 with default parameter settings (http://atted.jp, accessed on 30 April 2022) [13].

### 2.5. Expression Analysis of AtVPEs upon Drought Treatment

Total RNA was isolated via RNeasy Plant Mini Kit (Qiagen, Hilden, Germany) following the protocol described in [14]. First-strand cDNA was converted from 1 μg of the isolated total RNA using QuantiNova Reverse Transcription Kit (Qiagen, Germany). The expression profile was assessed via RT-qPCR analysis. Real-time PCR was performed with Bio-Rad CFX96 system (Bio-Rad, Hercules, CA, USA) with QuantiNova SYBR Green PCR (Qiagen, Germany) following the protocol as described in [15]. The primers (Supplementary Figure S5) were designed using Primer Blast from the National Center for Biotechnology Information (NCBI) and synthesized by Integrated DNA Technologies (IDT, Coralville, IA, USA). Three biological replicates were tested with three technical replicates performed on each sample. The data were analyzed using Bio-rad CFX Manager 3.1 software. The relative expression levels (2^−ΔΔCT^) were calculated according to Livak’s method [16]. The reference genes used in this study were *GAPDH* and *Actin*.

### 2.6. Plant Water Status

Relative water content (RWC) of detached leaves was determined according to [17] by measuring the fresh weight (FW) at the end of the drought period, and dry weight (DW) was obtained after drying the samples at 75 °C for at least 24 h. Turgor weight (TW) was determined by subjecting the leaves to rehydration for 2 h, after drought treatments. The RWC was calculated as follows:

$$\mathrm{RWC}\left(\%\right)=\frac{\left(\mathrm{FW}-\mathrm{DW}\right)}{\left(\mathrm{TW}-\mathrm{DW}\right)}\times 100$$

### 2.7. Biochemical Assays

Malondialdehyde (MDA) content was measured according to [18] with slight modifications. Powdered samples (0.2 g) were homogenized in 10 mL of 10% (*w*/*v*) trichloroacetic acid (TCA). Homogenate was centrifuged at 9660× *g* for 10 min. Then, 2 mL of the supernatant was mixed with 2 mL of 10% (*w*/*v*) TCA containing 0.6% (*w*/*v*) of thiobarbituric acid (TBA) and incubated at 100 °C for 20 min, and then quickly cooled on ice followed by centrifugation at 9660× *g* for 10 min. Absorbance at 532, 600, and 450 nm was measured using Jenway 7305 UV/Visible Spectrometer (Jenway, London, UK). The MDA content was calculated according to the formula MDA content (µM/gFW) = 6.45 (OD 532 − OD 600) − 0.56 OD 450.

Total proline content was obtained by homogenizing approximately 200 mg of fresh leaves in 2 mL of 3% sulfosalicylic acid and centrifuging at 3000× *g* for 20 mins. Then, 1 mL of supernatant was mixed with 1 mL of concentrated acetic acid and acid ninhydrin reagent prepared by dissolving 1.25 g of ninhydrin in 30 mL of 6 M H_3_PO_4_ and 20 mL of acetic acid. The mixture was boiled for 1 h and then added to 2 mL of toluene. The concentration of proline in the toluene fraction was determined by measuring the absorbance at 520 nm with a microplate reader (Synergy H1 Hybrid Reader, Biotek, Korea). Proline concentration was calculated with L-proline as the standard [19].

Total photosynthetic pigment content was determined by homogenizing 100 mg of plant leaf samples together with 2 mL of 80% (*v*/*v*) of acetone for 1 min in the dark. The homogenate was then centrifuged at 400× *g* for 5 min and the supernatant was collected up to 12.5 mL. The absorbance was recorded at 470, 646.8, and 663.2 nm. The concentration of the photosynthetic pigments (chlorophyll a, chlorophyll b, total chlorophyll, and carotenoids) was estimated according to [20] and expressed in mg/gFW. 

Total soluble sugar content was estimated according to [21] with slight modifications. In brief, 0.1 g of powdered samples were extracted twice in 2 mL of 90% (*v*/*v*) ethanol by incubating the samples at 60 °C for 1 h. After each extraction, the samples were centrifuged at 419× *g* for 5 min. Then, 1 mL of supernatant was mixed with 1 mL of 5% (*v*/*v*) phenol together with 5 mL of concentrated sulphuric acid. The mixture was cooled at room temperature before absorbance was recorded at 495 nm spectrophotometrically. The amount of soluble sugars was calculated against a glucose standard and expressed in mg/gFW.

### 2.8. Statistical Analysis

All data presented were the average ± standard deviation (SD) of three biological replicates. Student’s *t*-test was applied to evaluate the level of significant differences at *p* < 0.05 between the different treatments using the SPSS v.20 software (IBM Corp., Armonk, NY, USA).

## 3. Results

### 3.1. Analyses of Cis-Elements in the Promoter and Co-Expression Network of αVPE

**Figure 1:**
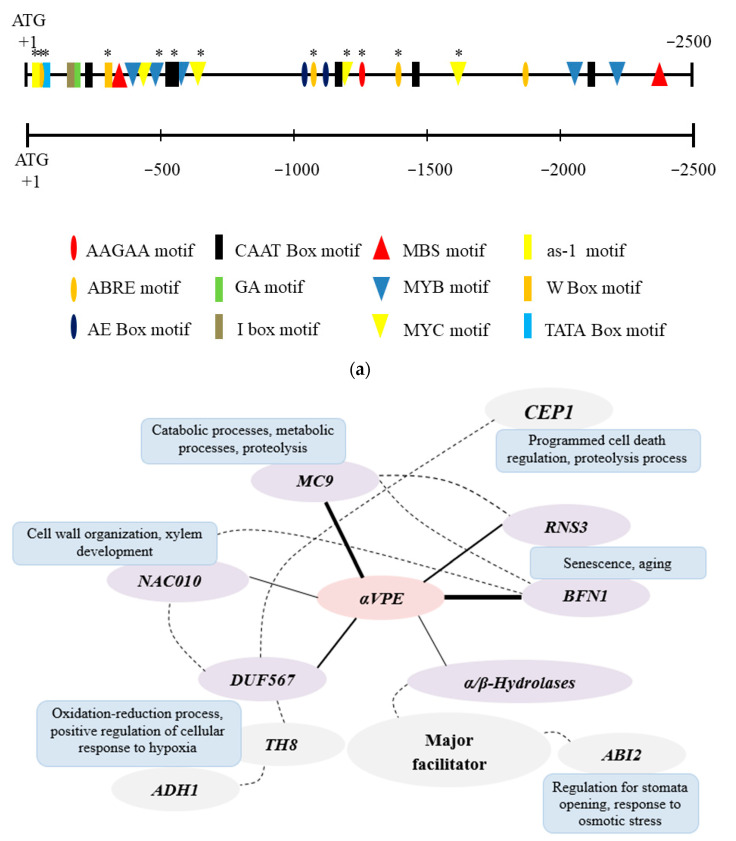
Predicted cis-element analysis in the promoter region of *αVPE* and co-expression networks for *αVPE* assembled from transcriptome data in ATTED-II with default parameters. (**a**) Promoter sequences (2.5 kb) of *αVPE* were analyzed by PlantCARE and PLACE Web Signal Scan. Different colour and shape boxes stand for different cis-elements. Asterisk symbol represents the cis-element in an inverted direction on the promoter region. (**b**) Bolder lines show a direct connection with *αVPE*, while dotted lines show an indirect connection of co-expressed genes with *αVPE*.

The promoter sequence analysis of *αVPE* using PlantCARE and PLACE showed a number of significant cis-elements (Figure 1). For example, AAGAA and AE Box are related to pollen-specific activation [22,23]. Dehydration-responsive elements such as ABRE, MBS, MYC, and MYB were identified in the promoter sequence [1,23]. The GA, I Box, and as-1 Box were identified as light-responsive elements [5]. The W Box was found to be a wounding-related element [23]. Drought-related cis-elements such as ABRE, MBS, MYB, and MYC motifs were frequently found in the *αVPE* promoter, indicating its involvement in the drought-related mechanism. An interaction network of the *αVPE* gene was constructed using ATTED-II version 6.1 with default parameter settings using microarray and RNA-seq datasets. From the networks, six genes including *bifunctional nuclease 1* (*BFN1*), *metacaspase 9* (*MC9*), *domain containing protein 10* (*NAC010*), *domain of unknown function 567* (*DUF567*), *ribonuclease 3* (*RNS3*), and *α/β-hydrolases* (Figure 1) were directly connected and co-expressed with the *αVPE* gene.

### 3.2. Expression Profile of the AtVPE Gene Family towards Drought Stress

**Figure 2:**
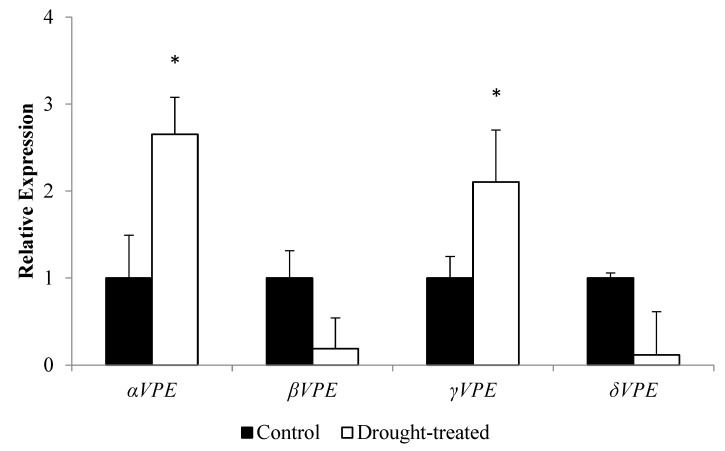
Normalised relative gene expression of the *AtVPE* family (*αVPE*, *βVPE*, *γVPE*, and *δVPE*) in WT upon drought treatment. Data indicate the mean (±SD) of three biological replicates. Asterisk symbol represents significant difference at *p* < 0.05 compared with the control.

Quantitative real-time PCR was performed on WT after drought treatment to examine the transcription level of the *AtVPE* gene family. Among the four *VPE* genes, *αVPE* and *γVPE* were upregulated (2.7-fold and 2.1-fold, respectively), whereas *βVPE* and *δVPE* were downregulated (0.19-fold and 0.12-fold, respectively) (Figure 2).

### 3.3. Morphological and Physiological Responses of Both Wild Type and Alpha Mutant towards Drought Stress

**Figure 3:**
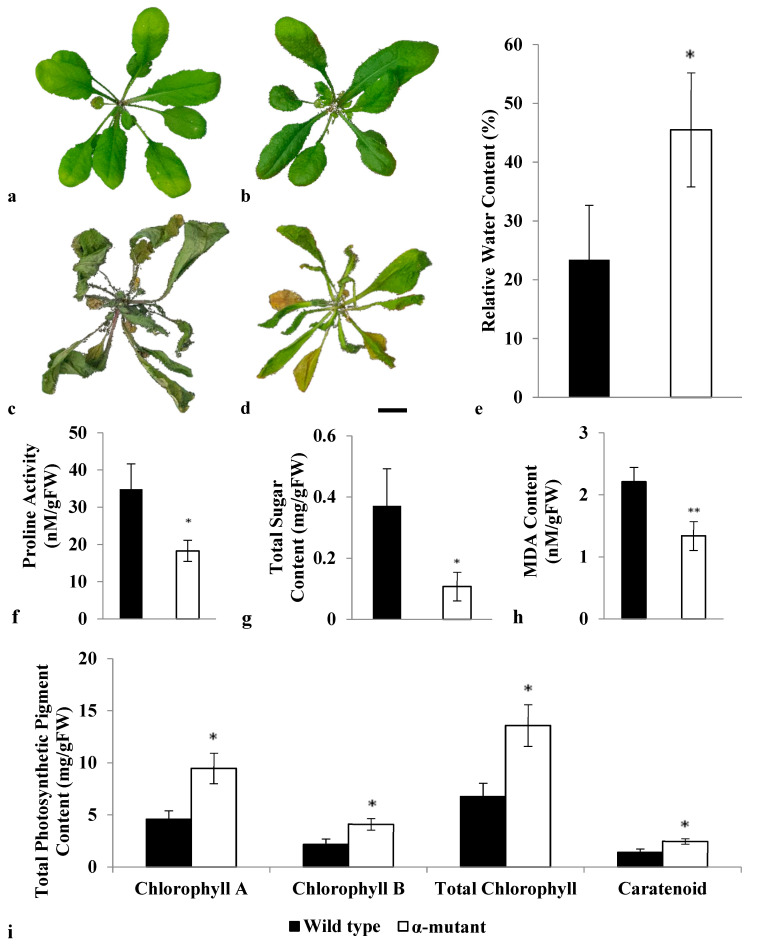
Effects on WT and alpha mutant upon drought treatment. Morphology of (**a**) WT before drought treatment and (**b**) alpha mutants before drought treatment. (**c**) WT after drought treatment. (**d**) Alpha mutants after drought treatment. (**e**) RWC (%) in leaves of both WT and alpha mutants after drought treatment. (**f**) Total proline activity was measured in nM/gFW. (**g**) Total sugar content was measured in mg/gFW. (**h**) Total MDA content was measured in nM/gFW. (**i**) Total photosynthetic pigment content was measured in mg/gFW. The results indicate the mean (±SD) of three biological replicates. Asterisk symbol represents a significant difference at *p* < 0.05 compared with wild type. Double asterisk symbols represent significant difference at *p* < 0.01 compared with WT.

To determine *αVPE* involvement in drought response, a loss of function study was carried out by comparing the morphological and physiological responses between both WT and alpha mutants after drought treatment (Figure 3). The alpha mutants’ leaves remained shiny and greenish compared with the WT after drought treatment (Figure 3). The alpha mutants’ leaves recorded 22% higher RWC than WT (Figure 3). The proline activity, total sugar content, total MDA content, and total photosynthetic pigment content in alpha mutants and WT after drought treatment are summarized in Figure 3. The alpha mutants (18.28 ± 2.78 nM/gFW) showed lower proline activity after drought treatment compared with WT (34.82 ± 6.84 nM/gFW). The sugar content in alpha mutants (0.11 ± 0.04 mg/gFW) was lower than in WT (0.37 ± 0.12 mg/gFW) after water withholding. The MDA content was reduced in alpha mutants (1.34 ± 0.18 nM/gFW) compared with WT (2.21 ± 0.23 nM/gFW). On the flip side, total photosynthetic pigments such as total chlorophyll pigments and carotenoid were higher in alpha mutants (13.60 ± 2.01 mg/gFW and 2.47 ± 0.26 mg/gFW, respectively) than WT (6.75 ± 1.27 mg/gFW and 1.40 ± 0.32 mg/gFW, respectively).

## 4. Discussion

Gene regulations at the transcriptional level are controlled by the cis-acting element motifs present in the promoter region [24]. Multiple cis-elements responding to different stresses suggest the possible functions of a gene [25,26]. In present study, the promoter regions of *αVPE* were analyzed using PlantCARE and PLACE to predict the cis-elements of the gene, whereby the majority of the elements were found to be related to drought motifs (ABRE motif, MBS motif, MYB motif, and MYC motif) [27,28], suggesting that *αVPE*, a type of vegetative VPE, is involved in the drought-related mechanism in *A. thaliana* plant development [9,29]. In addition, the presence of as-1 motif and I box motif [27] suggests that *αVPE* might involve a light-responsive mechanism.

To determine the possible functions of *αVPE*, a co-expressed network was constructed (Figure 1) and the co-expressed genes were further examined with their related mechanisms. *BFN1* and *RNS3* genes, co-expressed with *αVPE*, were found to be associated with plant developmental PCD and are involved in senescence and aging processes in *A. thaliana* [30]. The *MC9* was known to be involved in cell death regulation during plant immune response and plant vascular development [31]. *NAC010* that is co-expressed with *αVPE* was found to be associated with cell wall organization and xylem development, especially in secondary cell wall thickening [32]. Another co-expressed gene, *DUF567*, is known as one of the basal-defense-related genes [33] and its co-expressed genes, *TH8*, and *ADH1*, were involved in the oxidation-reduction processes and positive regulation of cellular response to hypoxia [34,35]. *DUF567* was reported to be co-expressed with *CEP1* for PCD regulation in *A. thaliana*, while *α/β-hydrolases* were found to initiate physiological responses to stomata opening and osmotic stress. Based on the functions of co-expressed genes, we believed that *αVPE* has a crucial role in regulating drought stress in *A. thaliana*.

For further elucidation of the role of *αVPE* in *A. thaliana* in response to drought, the expression profiles (Figure 2) for all *AtVPE*s after drought treatment were determined. Among the *AtVPE* gene family, *αVPE* achieved 2.7-fold (the highest) upregulation followed by *γVPE* (2.1-fold). A previous study showed that *αVPE* and *γVPE* shared the same evolutionary ancestor—angiosperm [29]. Consistent with the study of [29], our analysis also showed that *αVPE* and *γVPE* shared the same node in the phylogenetic tree of the *AtVPE* gene family (Figure S3). Besides, *αVPE* and *γVPE* have amino acid sequence similarity up to 81.80% (Figure S3). The majority of the cis-elements found on *γVPE* promoter also possess drought-related functions (Figure S1). Most of the co-expressed genes together with *γVPE* were reported to be involved in the osmotic regulation mechanism (Figure S4). Furthermore, a previous study performed by [36] reported that *γAtVPE* was involved in osmotic regulation of *A. thaliana* via stomatal movements. Therefore, we postulated that *αVPE*, which is nearly as congruent as *γVPE*, might also post a similar mechanism in stomata opening and closing, and both *αVPE* and *γVPE* will compensate each other when *A. thaliana* is under water deficit conditions.

The loss of function study showed that alpha mutants were recorded as greener than WT (Figure 3). Conversely, WT showed more sagging and yellow-brown leaves (Figure 3). This is because the drought regulation was interrupted in alpha mutants, leading to fewer drought effects on morphology. In addition, a previous study showed that a higher protein content and lower cysteine proteases activity were recorded in αVPE mutant plants upon drought treatment [37]. Hence, *αVPE* might negatively regulate drought tolerance in *A. thaliana*. The RWC of both alpha mutants and WT after drought treatment was taken as an evaluation of water status. Drought-sensitive plant species will have a relatively low water content compared with drought-tolerant plant species [38]. The leaves of alpha mutants contained more water as compared with WT (Figure 3), indicating that alpha mutants were more tolerant to osmotic stress owing to the absence of the drought regulator, *αVPE*. During water shortage, plants anticipate themselves with essential stress tolerance by regulating their cellular, physiological, and molecular mechanisms [39]. This is crucial to ensure they are prepared to survive in this extreme environment. A typical plant will produce more osmoregulator for better osmotic adjustment in response to drought [39]. Proline, an osmoprotectant, and a stress-responsive amino acid, was induced in plants that experienced drought stress [40]. In our study, alpha mutants showed reduced proline activity (1.8-fold), indicating they were less affected by water deficit conditions compared with WT (Figure 3). The results were in line with the study conducted by [41], suggesting that proline accumulation represents stress-induced damages as observed in drought-sensitive plants. Elevated proline activity at a low water potential will help in reducing impairment caused by ROS to protect cell membrane stability [42]. A commonly known osmolyte, sucrose, is also essential in counteracting osmotic stress in plants [39]. Similar to proline, accumulation of soluble sugar under drought conditions can help in promoting cell recovery and stabilizing subcellular structure [43]. In our study, alpha mutants contained a lower sucrose concentration compared with WT (Figure 3). This indicated that alpha mutants are more drought-tolerant compared with WT. The authors of [44] also concluded that the effects of water deficit conditions on plants will lead to photosynthesis inhibition, retarded growth, and sucrose accumulation in leaves. The MDA content was measured to examine the level of lipid peroxidation that caused membrane damage in both alpha mutants and WT. When a plant was under stress, ROS will be produced and leads to lipid membrane oxidation [45,46]. After drought treatment, WT exhibited a higher MDA content than alpha mutants (Figure 3). Higher MDA contents in plants reflect a higher level of stress imposed on the plants. This indicates that WT undergoes more drought stress than alpha mutants. In addition, lower photosynthetic pigments were recorded in WT as compared with alpha mutants (Figure 3). This is because the photosynthesis efficiency was affected under water deficit conditions in plants, which led to a reduced level of photosynthetic pigments [47]. With these physiological changes, we confirmed that *αVPE* negatively regulates drought tolerance in *A. thaliana*.

## 5. Conclusions

In this study, the majority of drought-related cis-elements were found in the promoter region of *αVPE* such as ABRE, MBS, MYC, and MYB. Similarly, the co-expression network also revealed that genes interacting with *αVPE* were involved in drought-regulation-related functions. In addition, *A. thaliana* under drought treatment recorded an upregulation of *αVPE* expression. The loss of function study showed that alpha mutants have better drought stress tolerance, with reduced proline, sugar, and MDA contents, coupled with an increase in RWC and photosynthetic pigments. Taken together, we successfully provided evidence that *αVPE* is a negative regulator of the drought tolerance mechanism in *A. thaliana.*

## Data Availability

All data generated or analyzed during this study are included in this published article. Additional data will be made available upon reasonable request.

## Supplementary Materials

The following supporting information can be downloaded at https://www.mdpi.com/article/10.3390/biology12030430/s1. Figure S1: Predicted cis-element analysis in promoter regions of *βVPE*, *γVPE*, and *δVPE*; Figure S2: Genotyping on the T-DNA insertion line of α-mutant *Arabidopsis thaliana* using LP, BP, and RP primer combination; Figure S3: Sequence analysis on *AtVPE* genes; Figure S4: Co-expression networks for *γAtVPE* assembled from transcriptome data in ATTED-II with default parameter; Figure S5: List of primers.
