# Supplementary material for: Promoter Cis-Element Analyses Reveal the Function of αVPE in Drought Stress Response of Arabidopsis
Source: Biology (Basel). 2023 Mar 10;12(3):430. doi: 10.3390/biology12030430 (PMC10045073; doi:10.3390/biology12030430)
Supplement: Supplementary file 1 [file biology-12-00430-s001.zip › biology-2185165-supplementary.pdf]

Supplementary Data

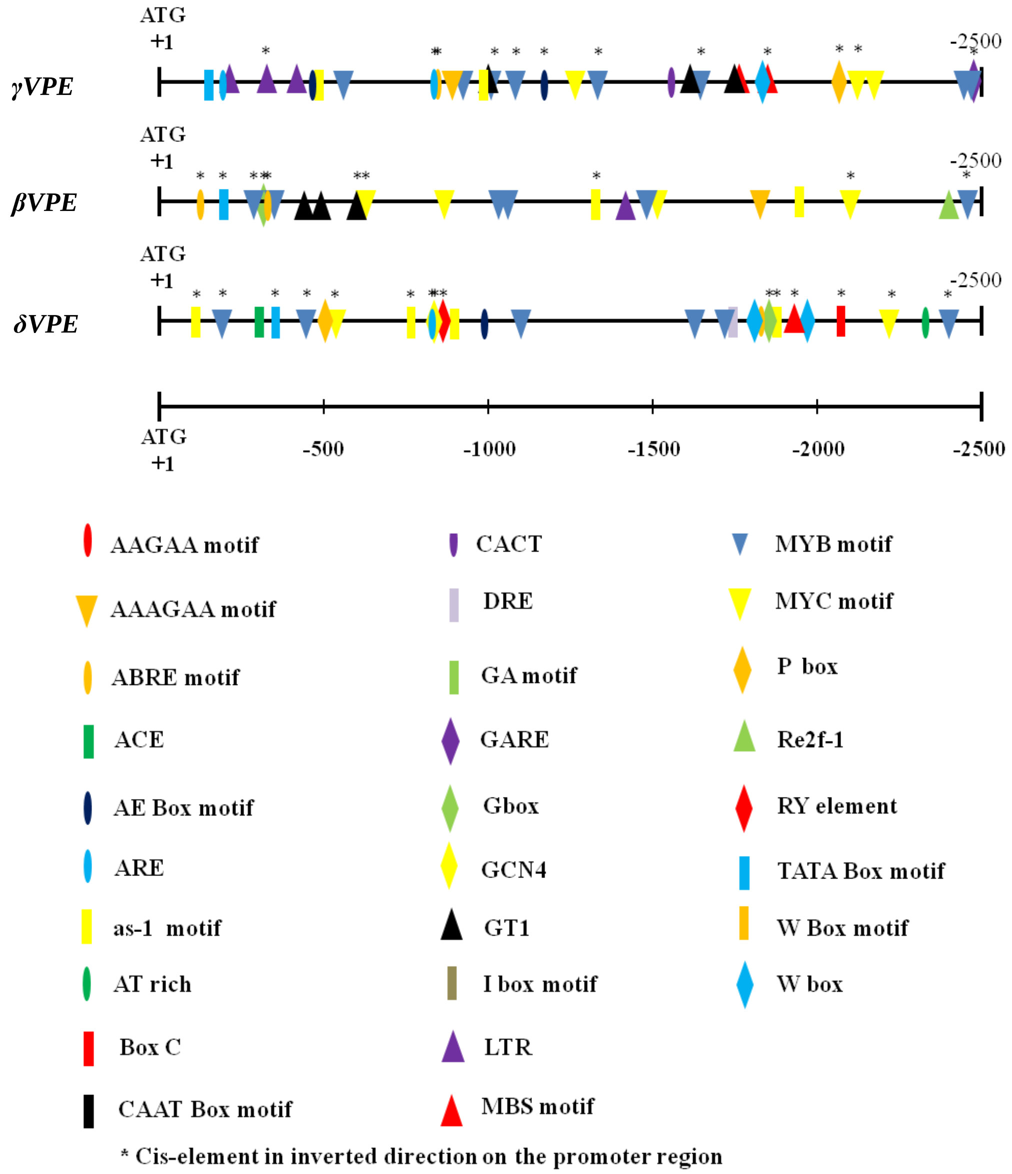

Figure S1 Predicted cis-element analysis in promoter regions of *βVPE*, *γVPE* and *δVPE*. Promoter sequences (2.5kb) of genes were analyzed by PlantCARE and PLACE Web Signal Scan. Different colour and shape boxes stand for different cis-elements. Asterisk symbol represented the cis-element in inverted direction on the promoter region.

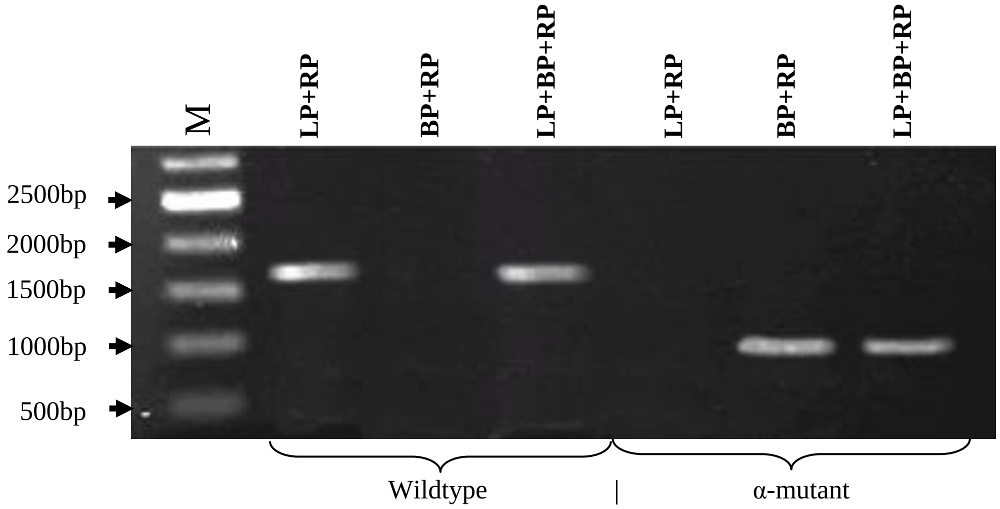

Figure S2 Genotyping on the T-DNA insertion line of α-mutant *Arabidopsis thaliana* using LP, BP and RP primer combination. LP and RP represent the left and right flanking priming sites within a VPE open reading flame. BP represents the priming site within the inserted T-DNA. LP+RP combination amplifies portion of the gene locus with or without T-DNA insertion while BP+RP produce a band only when the T-DNA is inserted within the locus. Lane M: Vivantis 1kp DNA Ladder was used. Lane LP+RP: Primers of LP and RP were used. Lane BP+RP: Primers of BP and RP were used. Lane LP+BP+RP: Primers of LP, BP and RP were used.

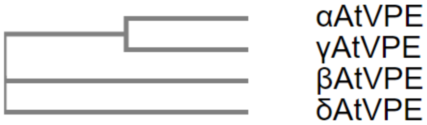

a

	**α*At*VPE**	**β*At*VPE**	**γ*At*VPE**	**δ*At*VPE**
**α*At*VPE**	100.00%	58.35%	81.80%	50.88%
**β*At*VPE**	58.35%	100.00%	60.17%	49.13%
**γ*At*VPE**	81.80%	60.17%	100.00%	50.43%
**δ*At*VPE**	50.88%	49.13%	50.43%	100.00%

b

Figure S3 Sequence analysis on *AtVPE* genes. (a) A phylogenetic tree of *AtVPE* gene family was performed by The European Bioinformatics Institute (EMBL-EBI), based on a ClustalW alignment and clustered by Neighbour-joining without distance corrections. (b) Percent Identity Matrix for *AtVPE* gene family was performed by Clustal 2.1 and measured in percentage (%) compared with each other.

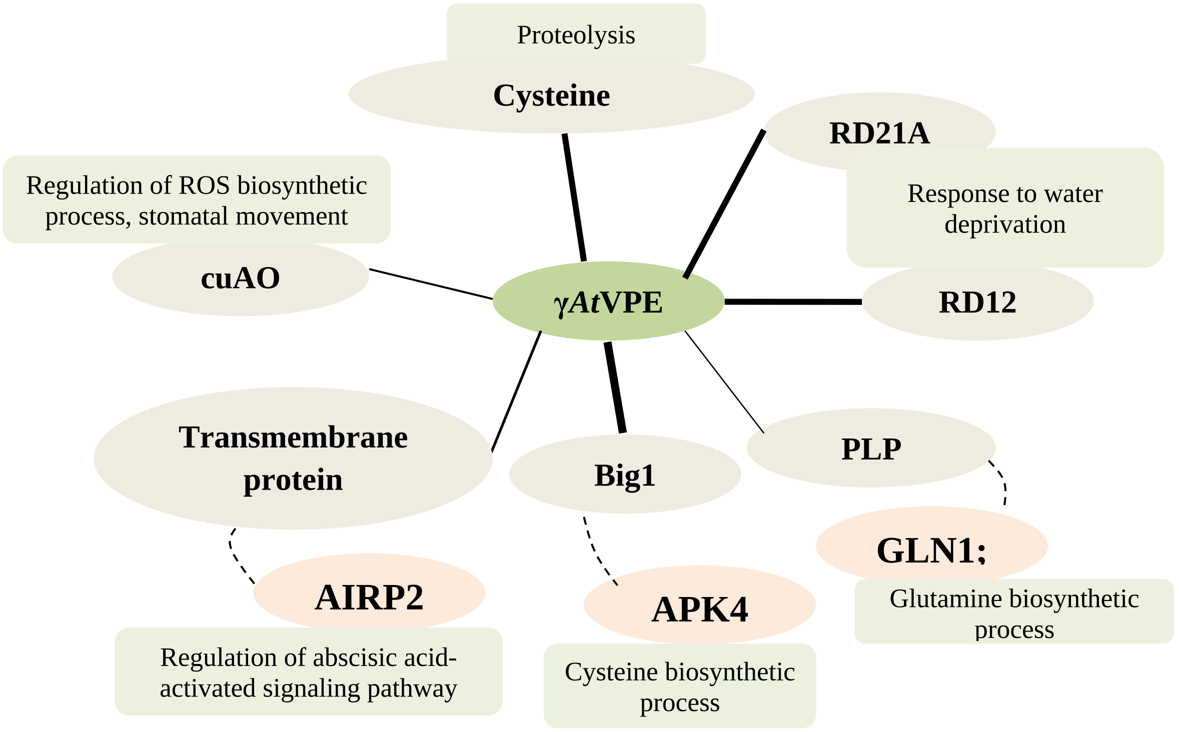

Figure S4 Co-expression networks for γ*AtVPE* assembled from transcriptome data in ATTED-II with default parameter. Bolder lines show direct connection with γ*AtVPE* while dotted lines show indirect connection of co-express genes with γ*AtVPE*.

**Primer Name**	**Primer Sequence (5’ to 3 ‘)**	**Annealing Temperature (°C)**
αVPE	FW: GGAGGCTTGTGAATCTGGAA<br>RV: TTAAGGCAGTCCCAATCGTC	60.0
βVPE	FW: GATTCTTATGCCGACAGAGG<br>RV: CCTGGTGTCTGTAGTTTCCA	60.0
γVPE	FW: AGTGGGAAGGTTGTGGATAG<br>RV: CTTTTCCCAAAAATGAACAAG	60.0
ΔVPE	FW: TGTACCAGAGACTTCTCATGTATGC<br>RV: TGCTTCAAGACTCTAGTTAATAGCT	60.0
GAPDH	FW: TTACGAAGGCGGTGTTTTTC<br>RV: GGCGAGGCGTGTATACATTT	60.0
Actin	FW: AGAGATTCAGATGCCCAGAAGTCTTGTTCC<br>RV: TATGATGAGGCAGGTCCAGGAATCGTT	60.0

Figure S5 List of Primer
